# From Oncogenic Signaling Pathways to Single-Cell Sequencing of Immune Cells: Changing the Landscape of Cancer Immunotherapy

**DOI:** 10.3390/molecules26082278

**Published:** 2021-04-14

**Authors:** Afshin Derakhshani, Zeinab Rostami, Hossein Safarpour, Mahdi Abdoli Shadbad, Niloufar Sadat Nourbakhsh, Antonella Argentiero, Sina Taefehshokr, Neda Jalili Tabrizi, Omid Kooshkaki, Reza Vaezi Astamal, Pankaj Kumar Singh, Nima Taefehshokr, Nazila Alizadeh, Nicola Silvestris, Behzad Baradaran

**Affiliations:** 1 Immunology Research Center, Tabriz University of Medical Sciences, Tabriz 51656-65811, Iran; afshin.derakhshani94@gmail.com (A.D.); abdoli.med99@gmail.com (M.A.S.); sinataefehshokr@gmail.com (S.T.); jalili.t.neda@gmail.com (N.J.T.); rezavaezi72@gmail.com (R.V.A.); alizadeh_imm@yahoo.com (N.A.); 2 IRCCS Istituto Tumori “Giovanni Paolo II” of Bari, 70124 Bari, Italy; n.silvestris@oncologico.bari.it; 3 Student Research Committee, Birjand University of Medical Sciences, Birjand 97178-53577, Iran; zeinabrostamy73@gmail.com (Z.R.); omidkoshki@gmail.com (O.K.); 4 Cellular & Molecular Research Center, Birjand University of Medical Sciences, Birjand 97178-53577, Iran; safarpour701@yahoo.com; 5 Student Research Committee, Tabriz University of Medical Sciences, Tabriz 51666-14766, Iran; 6 Department of Biology, Islamic Azad University, Varamin-Pishva Branch, Tehran 33817-74895, Iran; Niloufarnourbakhsh1994@gmail.com; 7 Principal Research Technologist, Department of Radiation Oncology, Mayo Clinic, 4500 San Pablo Rd S, Jacksonville, FL 32224, USA; Singh.pankaj@mayo.edu; 8 Department of Microbiology and Immunology, Center for Human Immunology, The University of Western Ontario, London, ON N6A 5C1, Canada; n.taefehshokr@gmail.com; 9 Department of Biomedical Sciences and Human Oncology, University of Bari “Aldo Moro”, 70124 Bari, Italy; 10 Department of Immunology, Faculty of Medicine, Tabriz University of Medical Sciences, Tabriz 51666-14766, Iran

**Keywords:** cancer, tumor microenvironment, signaling pathways, single-cell omics, tumor-infiltrating immune cells, single-cell sequencing of immune cells, immune checkpoints

## Abstract

Over the past decade, there have been remarkable advances in understanding the signaling pathways involved in cancer development. It is well-established that cancer is caused by the dysregulation of cellular pathways involved in proliferation, cell cycle, apoptosis, cell metabolism, migration, cell polarity, and differentiation. Besides, growing evidence indicates that extracellular matrix signaling, cell surface proteoglycans, and angiogenesis can contribute to cancer development. Given the genetic instability and vast intra-tumoral heterogeneity revealed by the single-cell sequencing of tumoral cells, the current approaches cannot eliminate the mutating cancer cells. Besides, the polyclonal expansion of tumor-infiltrated lymphocytes in response to tumoral neoantigens cannot elicit anti-tumoral immune responses due to the immunosuppressive tumor microenvironment. Nevertheless, the data from the single-cell sequencing of immune cells can provide valuable insights regarding the expression of inhibitory immune checkpoints/related signaling factors in immune cells, which can be used to select immune checkpoint inhibitors and adjust their dosage. Indeed, the integration of the data obtained from the single-cell sequencing of immune cells with immune checkpoint inhibitors can increase the response rate of immune checkpoint inhibitors, decrease the immune-related adverse events, and facilitate tumoral cell elimination. This study aims to review key pathways involved in tumor development and shed light on single-cell sequencing. It also intends to address the shortcomings of immune checkpoint inhibitors, i.e., their varied response rates among cancer patients and increased risk of autoimmunity development, via applying the data from the single-cell sequencing of immune cells.

## 1. Introduction

Cancer, in which genetic and epigenetic modifications have been implicated in its development, is the second leading cause of death worldwide [1,2]. Carcinogenesis is due to increased cell proliferation, resistance to apoptosis, genetic instability, angiogenesis, metabolism reprogramming, and cell migration [3,4]. Most of these changes are caused by dysregulated signaling pathways [5].

Advances in whole genome amplification and next-generation sequencing methods have paved the way for genomic analysis of single cells to detect genomic lesions in individual cancer cells. Although previous approaches could relatively characterize the properties of tumor cells, they could not precisely identify the genetic mutations in heterogeneous tumors [6]. The advances in single-cell sequencing technologies and their applications in cancer research can be considered as a revolution for our understanding of cancer development, tumor heterogeneity, and the tumor microenvironment [7].

This review aims to discuss the current knowledge about various pathways involved in cancers and the mechanisms that malignant cells use for immune evasion. Furthermore, this study also intends to highlight the obtained data from the single-cell analysis of tumor-infiltrating lymphocytes, which can facilitate the development of personalized cancer therapies for affected patients.

## 2. Tumorigenesis and Signaling Pathways

The overexpression of oncogenes and oncoproteins can promote abnormal signals leading to tumorigenesis [8]. Studies have shown that the activation of proto-oncogenes and the inactivation of tumor suppressor genes can contribute to tumorigenesis [9,10]. Compared to healthy cells, apoptosis evasion, continuous angiogenesis, proliferation, and migration, are the main characteristics of tumor cells [3,11,12]. Oncogenic mutations can affect the downstream nuclear targets of signaling pathways, e.g., enhancer of zeste homolog 2 (EZH2), cyclins, nuclear factor-kappa B (NF-κB), and Myc [1]. Besides, genomic lesions can inactivate tumor suppressors. The p53, which regulates cell proliferation and apoptosis, is mutated in about half of cancers [13,14,15]. The p16 gene (CDKN2A), which is a tumor suppressor gene, can inhibit cyclin-dependent kinase D. Most of the tumor suppressors function as negative cytoplasmic regulators, such as adenomatous polyposis coli protein (APC) and tensin homolog (PTEN). APC is a negative regulator of the Wnt pathway, and PTEN is a negative regulator of the phosphatidylinositol 3-kinase (PI3K)/protein kinase B (AKT) pathway [16,17].

Receptor tyrosine kinase (RTK)-Ras-extracellular signal-regulated kinase (ERK) signaling pathway is another dysregulated pathway with the highest median frequency of changes among all types of cancers [18,19]. Gain-of-function mutations, chromosomal rearrangements, and autocrine activation can stimulate the RTK-related pathways [20]. This pathway has been implicated in various cancers, e.g., melanoma, colorectal cancer, HER2-positive breast cancer, pancreatic cancer, isocitrate dehydrogenase 1 (IDH1)-wild-type glioma, lung adenocarcinoma, and thyroid carcinoma [1,21,22]. Furthermore, lung cancer, EBV-positive esophagogastric cancer, squamous cell carcinoma, and non-hypermutated uterine cancer have demonstrated high alterations of the PI3K pathway. The activation of phosphatidylinositol-4,5-bisphosphate 3-kinase catalytic subunit alpha (PIK3CA) and the inactivation of phosphoinositide-3-kinase regulatory subunit 1 (PIK3R1) are considered the major alterations of the PI3K pathway in breast cancer, head and neck cancer, and gynecological and gastrointestinal tumors [23]. Furthermore, the transforming growth factor-beta (TGF-β) signaling pathway is dysregulated in some cancers [24,25]. Pancreatic and gastrointestinal cancers have the highest alteration rate in the TGF-β pathway, whereas renal and brain cancers have almost no genetic alterations in this pathway [1]. Studies demonstrate that ovarian cancer and breast cancer cells highly depend on Myc for maintaining their tumoral growth, which can serve as a valuable target for the treatment of affected patients [26]. Besides, the N-myc amplification has been associated with the inferior prognosis of neuroblastoma patients [27,28].

Since G protein-coupled receptors (GPCRs) play pivotal roles in regulating cancer-related signaling pathways, they are now utilized as early diagnostic biomarkers for cancer [29]. GPCRs are the family of membrane proteins, regulating most cellular responses to hormones and neurotransmitters. Based on their sequence and structural similarity, GPCRs are generally classified into five families, including rhodopsin, secretin, glutamate, adhesion, and Frizzled/Taste2 [30]. These proteins have strongly hydrophobic transmembrane helix structures. Stimuli such as photons, small chemicals, ions, and protein ligands cause conformational changes in the GPCRs, which can be translated into the target cells by activating heterotrimeric G proteins [29]. Initial findings have indicated that GPCR dysregulation can be implicated in tumor development [31]. Consistent with these, recent studies have illustrated mutations in G proteins and GPCRs. Indeed, GPCRs play pivotal roles in tumor cell proliferation by activating Rho GTPases, cytoskeletal changes, angiogenesis, and metastasis. Moreover, GPCRs can give rise to an immunosuppressive tumor microenvironment for immune evasion of tumoral cells [29].

### 2.1. Cell Proliferation

Dysregulated cell proliferation is essential for cancer development. Indeed, modified expression and activation of cell-cycle-dependent proteins are the main culprits of dysregulated tumor proliferation [32]. Notch proteins, a family of type I transmembrane receptors, play substantial roles in tumor proliferation [33]. The Notch1 signaling mediated hypoxia/HIF-1α can stimulate cell proliferation in T-cell acute lymphoblastic leukemia (T-ALL) [34]. It has been reported that Jagged-2 (JAG2) can bind to Notch2 and stimulate tumor proliferation [35]. Moreover, Notch3 has been associated with tumor proliferation [36].

The activation of the PI3K/Akt/mechanistic target rapamycin complex I (mTORC1) pathway can regulate cell growth [37]. The Akt phosphorylates glycogen synthase kinase 3 (GSK3), cyclin D, and Myc and inhibits catalytic activity [38,39]. Moreover, Akt inactivates several cell cycle inhibitors, such as p21 (CIP1) and cyclin-dependent kinase inhibitor p27 (KIP1); however, the phosphorylated Akt can lead to 14-3-3 proteins sequestration in the cytoplasm [40]. Moreover, the Akt can inhibit the transcription factors of the forkhead box (FoxO), e.g., retinoblastoma-like protein 2 (RBL2) and p27, which can stimulate the phosphorylation of the mouse double minute 2 homolog (MDM2) and ubiquitin ligase to degrade p53 [41,42]. The Akt can also control multiple enzymes involved in the G2/M transformation of the cell cycle [43]. Besides, Akt can phosphorylate β-catenin, which results in its separation from cadherin cellular adhesion complexes and the promotion of β-catenin transcriptional activity [44]. As mentioned above, signaling pathways are the cornerstone of cell proliferation in cancers. Since cytokines and the RTK signaling can activate the signal transducer and activator of transcription 3 (STAT3) and NF-κB, they can activate the expression of Myc and cyclin D [45].

**Figure 1:**
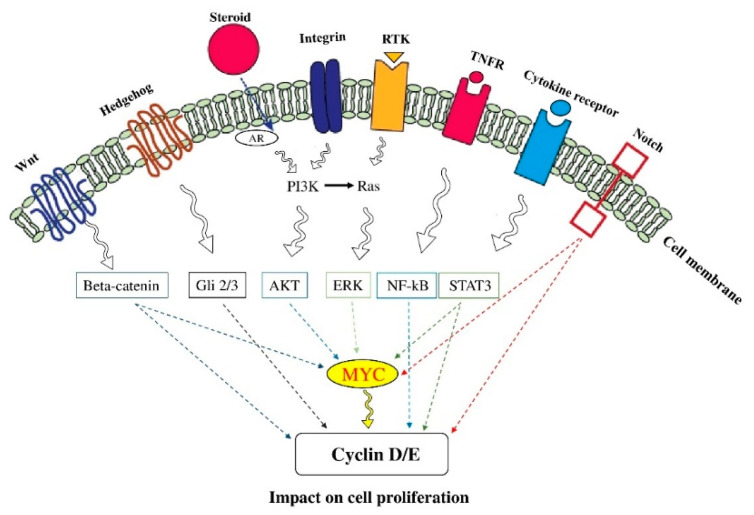
The signaling pathways of cell proliferation. Proliferation is an essential step in cancer development. The constitutive stimulation of signal transduction pathways can promote cancer development. Indeed, abnormal cell proliferation is a hallmark of most cancers and involves the modulation of multiple signaling pathways (see the main text for detail). The figure was produced using Servier Medical Art (http://smart.servier.com/) (assessed on 2 January 2020).

The role of tumor suppressors is critical to inhibit proliferative signals. Indeed, tumor suppressor mutations remove the brake and allow the tumoral cells to proliferate. The pRB directly inhibits the protein transcription of E2F via the phosphorylation of CDKs. In response to stress signals, the p53 blocks cell proliferation via inhibiting CDK activity and inducing CKIs. The CKIs, which can directly inhibit CDKs, can be inactivated via mutations in various cancers. The APC and Ras-GAP NF1, which inactivate the Wnt/β-catenin signaling by enhancing GSK3 phosphorylation, can inhibit the function of β-catenin [46]. Moreover, the Ras/PI3K/ERK pathway can lead to c-Myc stabilization via suppressing ubiquitylation [39]. Myc stimulates cell proliferation by regulating various cell proteins, e.g., CDKs, G1/S cyclins, and the cell cycle-driven E2F family transcriptional factors [47] (Figure 1).

### 2.2. Cell Survival

**Figure 2:**
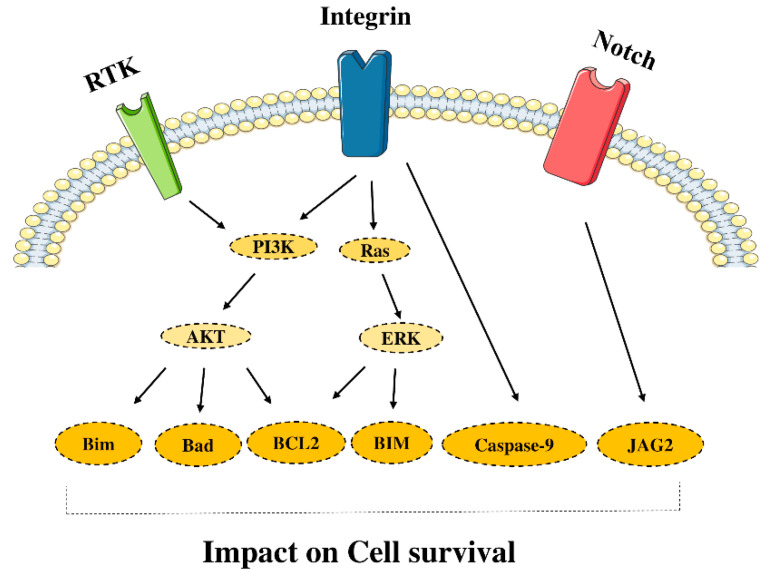
Signaling pathway and cell survival. Escape from cell death is one of the mechanisms to bypass anticancer treatments. The key players in preventing apoptosis are known as survival proteins. The figure was produced using Servier Medical Art (http://smart.servier.com/) (assessed on 2 January 2020).

Cell death serves as a homeostatic mechanism; however, mutations can dysregulate cell death signals in various cancers. For instance, the Notch signaling can stimulate cell survival by interacting between Jagged-2 (JAG2) ligand and receptor Notch2 in multiple cancers [48]. Moreover, studies have indicated that the Ras-ERK and PI3K-Akt pathways can control cell death in many malignancies [49]. The Akt also can interfere with the apoptotic signaling of death receptors. It inhibits pro-apoptotic B cell lymphoma 2 (Bcl2)-family member Bim and death ligands, e.g., Fas ligand (FasL) and TRAIL. Moreover, the Akt stimulates the X-linked inhibitor-of-apoptosis protein apoptosis inhibitor (XIAP) [50]. Furthermore, the Akt activates the NF-κB pathway, which regulates anti-apoptotic enzymes, i.e., Bcl2, BCLxl, and Mcl1 [50]. Moreover, the Akt can repress the p53-induced apoptosis via the degradation of p53 [41]. Bim, a pro-apoptotic BH3 member of the Bcl-2 family, can be regulated by MEK/ERK-mediated phosphorylation, which affects its binding to Bcl-2 family members and its turnover [51] (Figure 2). Besides, recent findings have indicated that c-Jun N-terminal protein kinases (JNKs), as the members of the MAPK family, can regulate tumor proliferation. However, JNK1 and JNK2 have opposing functions in pancreatic cancer cells. The inhibition of JNK2 has been associated with increased tumor proliferation of pancreatic cancer cells [52]. It has also been reported that JNK2 can protect p53 from MDM2-induced degradation, and the downregulation of JNK2 has been associated with the inferior prognosis of patients with bladder cancer who underwent cystectomy [53]. Therefore, the activation of JNK2 might be associated with the suppression of tumoral cells in pancreatic and bladder cancers.

### 2.3. Cell Metabolism

Metabolism dysregulation, e.g., the mutations of IDH1 and IDH2, can increase metabolites in cancer cells [54]. The inhibition of tumor suppressor genes and activation of oncogenes can dysregulate the metabolic pathways. For instance, the PI3K-Akt pathway can reprogram the metabolism [55]. The Akt regulates the transport of glucose, which can promote glycolysis [56]. Indeed, the Akt2 regulates the transcription, accumulation, and traffic of glucose transporter 1 (GLUT1) [57,58,59]. In line with these, it has been reported that the activation of Akt can substantially stimulate glycolysis in tumoral cells [60].

**Figure 3:**
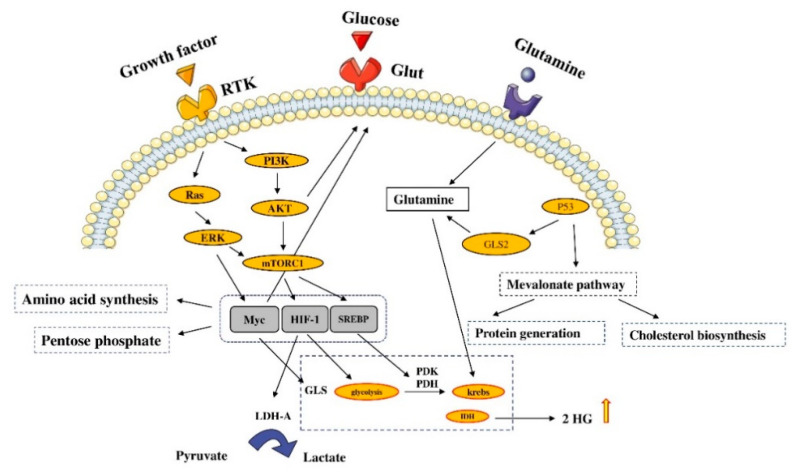
Signaling pathways and cell metabolism. Metabolism dysregulation is a common phenomenon in cancer cells. Different mutations, such as isocitrate dehydrogenase 1 (IDH1) and 2 (IDH2) mutations, can lead to the increased survival of cancer cells. The figure was produced using Servier Medical Art (http://smart.servier.com/) (assessed on 2 January 2020).

The mTORC1 increases the synthesis of the hypoxia-inducible transcription factor (HIF-1), which promotes lactate dehydrogenase (LDH-A) and glycolytic enzymes [61,62]. Furthermore, the mTORC1 regulates the localization of proteins involved in amino acid metabolism. It has been reported that mTORC1 activation can lead to the inactivation of the eIF4E-binding proteins (4E-BPs), which leads to increase protein synthesis [63]. The sterol-response-element-binding protein 1 (SREBP) transcription factor, activated by mTORC1, can increase lipid synthesis [64,65,66]. The inhibition of SREBP suppresses tumor growth by uncoupling fatty acid synthesis from desaturation. Furthermore, the Ras-ERK signaling has essential roles in glucose uptake, glycolysis, the pentose phosphate cycle, producing glutamine transporter, and glutaminase enzyme (GLS) [67,68,69]. Cancer cells demonstrate an increased level of glycolysis and glutaminolysis. Glycolysis can produce energy, and glutaminolysis can provide biosynthetic precursors for cancer cells [70]. Despite the higher rate of glycolysis in cancer cells than in non-cancer cells, many cancer cells generate an alternative and less active type of pyruvate kinase isozyme, i.e., pyruvate kinase muscle isozyme M2 (PKM2) [71]. The PKM2 is a limiting glycolytic enzyme that mediates the final step in glycolysis. The Ras-ERK and PI3K-Akt pathways can alter the activity of the PKM2 [72]. Besides the tumor-suppressive function of p53 on tumoral cells, p53 has pivotal roles in metabolic reprogramming. Indeed, p53 can downregulate the expression of GLUT1, GLUT3, and GLUT4 [73,74]. Additionally, p53 can repress glycolysis, which is partially due to the inhibitory effect of p53 on hexokinase 2 [75]. Moreover, p53 can inhibit the PI3K/Akt signaling pathway, leading to glycolysis suppression [76]. The p53 can regulate glutaminase 2 (GLS2), which is a crucial enzyme in converting glutamine to glutamate and lowering intracellular reactive oxygen species levels [77] (Figure 3). Recent findings have indicated that the gain-of-function mutations of p53 can increase glycolysis via upregulating the expression of GLUT1 and promoting the function of hexokinase 2. Besides, gain-of-function mutations of p53 can stimulate the mevalonate pathway via the activation of SREBP [78,79].

### 2.4. Cell Migration

**Figure 4:**
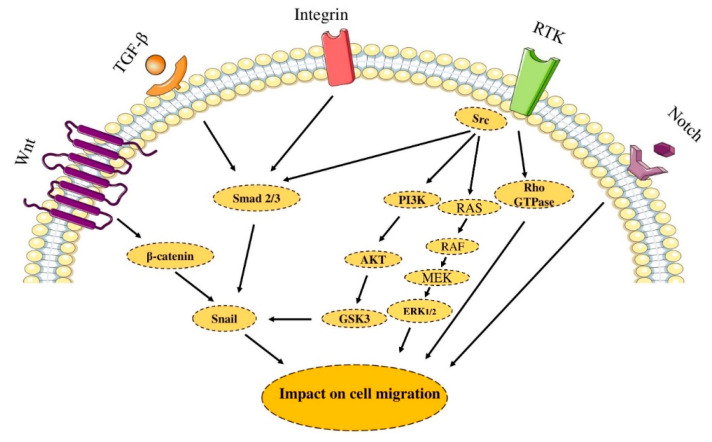
Signaling pathway and cell migration. Metastasis is a multistep process that includes migration and invasion of cancer cells. Migration is regulated by adhesion receptors, chemokines, growth factors, and other stimulators. The figure was produced using Servier Medical Art (http://smart.servier.com/) (assessed on 2 January 2020).

Cell migration is regulated by adhesion receptors, chemokines, and growth factors [80,81]. The Akt2 augments migration via regulating integrin activity and the epithelial–mesenchymal transition (EMT) process [82]. The EMT is associated with some physiological processes, e.g., wound healing, gastrulation, and morphogenesis splitting [83]. This type of development is regulated by various highly organized processes, including TNF, Notch, Wnt, TGF-β. The EMT is characterized by the decreased expression of E-cadherin, the loss of apical-basal polarity, the adoption of a fibroblast-like appearance, and the activation of stem-cell phenotypes. These modifications can augment the capacity of the cell to invade other tissues. It has been reported that the PI3K-Akt and Ras-ERK pathways can promote EMT when stimulated along with other involved pathways, e.g., the Notch, Wnt, and TGF-β signaling [84]. Some transcription factors, e.g., SLUG, SNAIL, ZEB, and TWIST, also play crucial roles in enhancing EMT. For instance, the Akt can phosphorylate IκB kinases, which can stimulate SNAIL [85]. Therefore, Akt activation can stimulate SNAIL, which leads to EMT activation. Furthermore, the Akt2 can phosphorylate the HNRNP E1, a protein that stimulates translational elongation in EMT-promoting transcripts [86]. Moreover, activator protein 1 (AP-1), regulated by the Ras-ERK pathway, can stimulate EMT-promoting transcription factors. This involves the stimulation of E-cadherin, fibronectin, vimentin, integrin heterodimers, e.g., aVb6 and a5b1, and cytokeratin [87,88]. The Notch signaling can also promote tumor migration via Notch3 activation [89]. Collectively, the aberrant activation of PI3K-Akt and Ras-ERK pathways can play substantial roles in tumor migration (Figure 4).

### 2.5. Cell Polarity

Polarity proteins are essential for maintaining tissue architecture. Three complexes have essential functions in polarity control, i.e., the complex of Scribble, Par, and Crumbs. The aberrant stimulation of the PI3K-Akt and Ras-ERK signaling pathways have been associated with the dysregulation of cell polarity. For instance, Scribble inhibits ERK activity by functioning as a scaffold to link it to the protein phosphatase PP1G [88,90]. Additionally, the loss of Scribble stimulates H-Ras activation, which promotes tumor development [91,92]. Moreover, the loss of the Par3 can dysregulate cellular polarity via the JAK/STAT3 signaling [93,94]. This stimulates the expression of matrix metalloproteinase 9 (MMP9), resulting in tumor invasion [95,96].

### 2.6. Cell Differentiation

Among cancers that disrupt the equilibrium between cell differentiation and proliferation, acute promyelocytic cancer is a poorly differentiated one. The fusion of PML and a receptor of retinoic acid (RAR) might be the main culprit [97]. Since the PML-RAR fusion inhibits RAR genes, this can block the RAR signal. Additional mutations can result in the uncontrolled proliferation of undifferentiated myeloblasts [98]. In colon crypts, the Wnt/β-catenin signaling can keep enterocytes in an undifferentiated state [98]. As cells migrate to the luminous intestine surface, APC can induce β-catenin degradation. However, the APC tumor suppressor mutation can save β-catenin. Developmental signals may also enhance cancer development because they can promote the proliferation of cells. For instance, mutations that promote the Notch pathway can lead to acute lymphocytic leukemia via promoting the cell cycle and suppressing T cell apoptosis [99]. In medulloblastoma and basal cell carcinoma, the mutations of patched receptors can stimulate the Hedgehog signaling pathway, resulting in increased cell proliferation [100]. Indeed, the Hedgehog signaling is upregulated in various malignancies via autocrine loops that can influence embryonic gastrointestinal tissues. The PI3K-Akt and Ras-ERK pathways are stimulated by fibroblast growth factor (FGF), insulin-like growth factor (IGF), and epidermal growth factor (EGF). For instance, the FGF4/8 can stimulate the Ras-ERK pathway to promote EMT [101]. Furthermore, the Notch signaling can affect cellular differentiation via Hes1 and glioma-associated oncogenes (Gli2) [89,102].

## 3. Cancer and Extracellular Matrix (ECM)

The tumor microenvironment comprises immune cells, fibroblasts, adipocytes, endothelial cells, and ECM [103]. Tumor cells and cancer-associated fibroblasts (CAFs) are responsible for producing the molecules of the ECM. Indeed, fibroblasts/myofibroblasts can infiltrate solid tumors and result in collagenous matrix accumulation in the tumor microenvironment [104]. ECM is considered a dynamic framework and plays focal roles in regulating the fate of tumors. Cellular phenotypes and molecular functions are dependent on signals from outside the cell, such as the interactions with the extracellular matrix [105]. Indeed, the duration of cancer mass is stemmed from the ECM components, e.g., collagens I, II, III, V, IX, and XI, heparan sulfate proteoglycans, and ECM-modifying enzymes [106].

Collagen, laminin, and fibronectin serve as ligands stimulating integrin development. In addition to tyrosine kinase FAK, the ECM signaling results in the activation of canonical processes, e.g., the PI3K-Akt and Ras-ERK signaling pathways. This scaffold connects integrins with cytoskeletal proteins, adapters, and enzymes, resulting in the transmission of signals from complex matrix adhesion [107]. Integrins are heterodimeric receptors that can lead to ECM remodeling and play a pivotal role in the tumor microenvironment. Transmembrane structures in the cytoskeleton are connected to both α and β subunits [108,109]. Since integrins are present on virtually all surfaces of nucleated cells, they are active in tumor cells and stromal cells [110,111]. CAFs are the major promoters of tumorigenic characteristics and can affect ECM remodeling. Besides, they can stimulate the survival, migration, and proliferation of the tumor cells. Moreover, the ECM functions as a reservoir of cytokines and growth factors, which can contribute to the bidirectional communication of the tumor cells and stroma [112,113]. Recent studies have also illustrated the cross-talk between CAFs and ECM can regulate the metastatic niche and chemoresistance [114].

### 3.1. Cell Surface Proteoglycans

**Figure 5:**
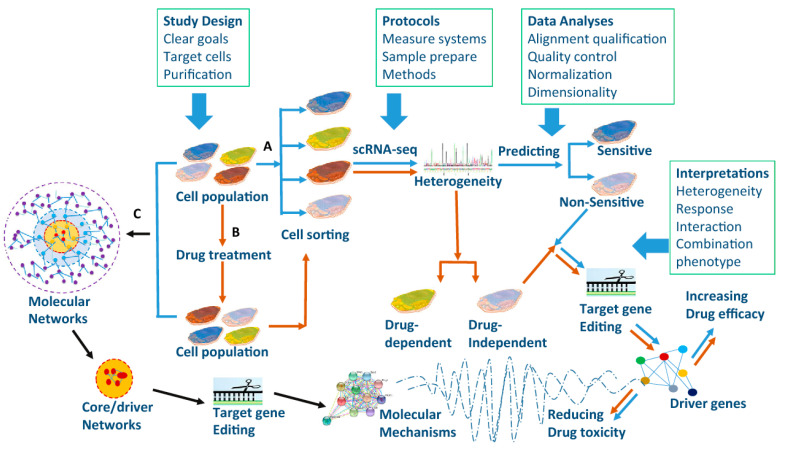
Single-cell RNA sequencing application in drug-response prediction. Targeted cell populations are selected from human organs/tissues and then one type of cell population can be sorted. Drug-dependent gene mutations and drug specificity of targeted genes can be defined after selected cells are treated with drugs (B, brown arrows). On the other hand, gene sequences and epigenetics of selected cells can be measured before the cell sorting (C, black arrows) to compare the cell population with single-cell sequences. Molecular mechanisms, drug efficacy, and toxicity of identified core/driver genes and networks can be validated by editing target genes. Reprinted with permission from ref. [157]. Copyright 2017 SpringerLink.

Studies have indicated that proteoglycans are a group of proteins that covalently connect to one or more glycosaminoglycan chains. Only a few types of glycosaminoglycans have been identified in mammals, e.g., keratan sulfate (KS), hyaluronan (HA), heparan sulfate (HS), and chondroitin sulfate (CS), which is closely-related dermatan phosphate (DS) [115]. The HA is not connected to a core protein and can be cross-linked with ECM proteins [116]. However, it is recognized as a critical ECM component for tumor biology [117] (Figure 5).

#### 3.1.1. The Classes of Cell Surface Proteoglycans

There are two main types of cell surface proteoglycan, i.e., syndecans and glypicans. Syndecans are connected to the cell membrane via a phospholipid anchor [118]. Moreover, ECM macromolecules with heparin-binding characteristics have been discovered in all types of matrixes. In wound healing processes, glycoproteins like fibrinogen, fibronectin, and Von Willebrand factor have critical roles [119]. On the other hand, thrombospondins, tenascins, and CTGF/Cyr61/NOV (CCN) genes can be expressed during tumorigenesis [120,121].

#### 3.1.2. CD44-Specific Signaling

CD44 has drawn particular attention because its various variants have been implicated in the development of multiple cancers [20,122]. The CD44v6 can function as a co-receptor for the c-Met or vascular endothelial growth factor where the ligand of the hepatocyte growth factor binds to both receptors [123]. Its downstream signaling can interact with the Ras-dependent signals, which results in increased invasion and actin cytoskeletal organization [124]. In response to the ECM modifications, the CD44v6 isoform can also activate the PI3K/Akt signaling pathway [125,126]. Moreover, CD44 is a major cancer stem cell marker in breast, prostate, pancreatic, and colon cancer [122,127].

#### 3.1.3. Syndecans and Cancer

Although there is little evidence indicating the roles of syndecans in tumor progression, several studies have revealed syndecans are altered in solid and hematopoietic cancers [128,129]. However, it is unclear whether these changes have critical roles in tumor progression or not. Nevertheless, syndecan-1 activation has been associated with myeloma development [130]. Furthermore, it has been demonstrated that syndecan-1 is necessary for the Wnt1 to promote tumor development, indicating its association with the β-catenin/TCF signaling [131].

## 4. Angiogenesis

Interleukin-8 (IL-8), FGF, vascular endothelial growth factor (VEGF), and platelet-derived growth factor (PDGF) have been implicated in angiogenesis. The PI3K-Akt pathway regulates angiogenesis and vessel stabilization [97,132,133]. Indeed, the PI3K-Akt signaling upregulates the HIF-1, a factor that can stimulate VEGF expression in cancer cells. The PI3K-Akt pathway also regulates other angiogenic factors, e.g., angiopoietins. The MMP9, which is a macrophage-secreted factor, is required for the VEGF-induced angiogenesis in cancers [134]. The thrombospondins-1,2 are active endogenous angiogenesis inhibitors. They prevent angiogenesis by directly antagonizing VEGF and affecting the development, growth, proliferation, and apoptosis of endothelial cells [135]. Indeed, the thrombospondins-1,2 are crucial inhibitors of angiogenesis and tumor development [136]. Therefore, thrombospondins inhibition might be an essential step for angiogenesis in different cancers [137].

The bone marrow-derived cell populations also regulate angiogenesis [138,139,140]. Macrophages, neutrophils, mast cells, and myeloid-lineage-derived precursors can induce cancer-related inflammation, which results in dynamic interaction with the angiogenic process in the tumor microenvironment [141]. Indeed, the bone marrow-derived progenitors migrate in the cancer milieu, participating in the endothelial and pericyte development [142,143,144,145,146].

## 5. Inflammation

Inflammatory cells can secrete FGF and EGF, which can initiate the PI3K-Akt and Ras-ERK signaling pathways in cancer cells. They also express colony-stimulating factor 1 (CSF1), which can promote EGF release [147]. Chronic inflammation has been implicated in cancer development. The notorious examples of chronic inflammation-induced malignancies are ulcerative colitis, chronic gastritis, and viral hepatitis, which can lead to the development of colorectal cancer, gastric cancer, and hepatocellular carcinoma, respectively. Besides, chronic inflammation can result in genetic instability. Indeed, the increased level of reactive oxygen species can lead to mutation and tumor initiation [148]. Under non-chronic inflammation, expression of proinflammatory cytokines such as IL-1 has been associated with tumor rejection; however, proinflammatory cytokines can lead to tumor development under chronic inflammatory conditions. It has been reported that IL-1 via activating the MyD88 and interleukin-1 receptor–associated kinase-4 (IRAK4) can stimulate the NF-κB pathway, which ultimately leads to the expression of proinflammatory cytokines [149]. Compared to normal tissues, macrophage infiltration is more predominant in prostatic intraepithelial neoplasia, which can upregulate the expression of cytokines, such as CXCL1 and CCL2. The expression of these macrophage-released cytokines has been associated with the activated ERK in the neoplastic cells [150]. Shi et al. have indicated that TNF expression is related to gallstone-induced gallbladder cancer [151]. Indeed, TNF-alpha might be a double-edged sword in chronic inflammatory conditions and cancer progression [152].

## 6. Single-Cell Sequencing

Although different pathways and genes have been identified in cancer development, the current treatment has not brought desired results. Indeed, applying high-throughput omics strategies for analyzing biological samples, e.g., transcriptomics, genomics, proteomics, and metabolomics, can further our knowledge of intra-tumoral heterogenicity [153,154] (Figure 5). During the last decade, the exponential development of high-throughput technologies, such as microarray and next-generation sequencing (NGS), has introduced new gene expression profiles in various diseases [155]. More recently, single-cell analysis allowed discovery of new dimensions that will possibly monitor the trajectories of distant cell lineage in tumor progression. The data produced via single-cell multi-omics uncovered the main biological processes, cellular heterogeneity mechanism, and resistance mechanism. Indeed, the insights obtained from single-cell analysis have allowed a deeper understanding of cancer and its microenvironment [156].

### 6.1. Dissecting Key Cellular and Molecular Functions in Cancers

Intratumor heterogeneity is a crucial characteristic of cancers. For example, in breast cancer, the characterization of this heterogeneity at a molecular level is a major challenge for efficient therapy. Investigating the gene expressions at the cellular level has provided new insights into tumor heterogeneity, which bulk RNA-seq could not investigate [158]. For instance, Roerink et al. integrated single-cell data with tumor organoid culture for studying intra-tumor heterogeneity of colorectal cancers. They found that cancer cells can have more somatic mutations than normal cells, and these mutations emerge mostly during final dominant clonal expansion. Their research has established a cancer evolution model by characterizing the architectures of the clonal and sub-clonal tumors and has identified the possible mechanisms of cancer progression. Indeed, these results can be an example of the unique ability of single-cell sequencing to classify heterogeneous cancer cells [159].

In 2020, Zhang et al. created scTPA, A web tool for single-cell transcriptome analysis of pathway activation signatures, (http://sctpa.bio-data.cn/sctpa), which is a web-based platform devoted to discovering pathway signatures and intepreting single-cell RNA-seq data. The abundance of high-quality selected biological pathways with various functional and taxonomic classifications was gathered manually, enabling the discovery of pathways according to research background and interests. The scTPA integrates four commonly used approaches to evaluate pathway activation profiles and provides versatile criteria for downstream analysis; scTPA offers an easy-to-use platform for accessing and uploading pathway activity scores, cell clustering, pathway signatures, and related gene expression [160].

### 6.2. Molecular Mechanisms of Drug Resistance

In cancer therapy, therapy resistance is a daunting challenge. Although chemotherapy has shown promising results in treating affected patients, some patients develop drug resistance and progress to metastatic steps [161]. In this regard, major research areas include clarifying the resistance mechanisms and discovering prognostic factors. For example, two scRNA-seq-based studies have identified that microphthalmia-associated transcription factor, AXL, and dopachrome tautomerase signatures are related to the RAF/MEK-inhibitor resistance in melanoma [162,163]. Furthermore, analyzing the scRNA-seq data of circulating tumor cells in prostate cancer has identified activated non-canonical Wnt signaling pathways in the resistance of androgen receptor inhibitors [164]. Kim et al. utilized the single-cell DNA and RNA sequencing in addition to bulk exome sequencing to clarify the cause of resistance of triple-negative breast cancer to chemotherapy. They investigated whether the resistance results from selecting rare pre-existing clones or the development of new genomic aberrations. The results indicate that resistant genotypes are pre-existing and adaptively chosen by neoadjuvant chemotherapy, and transcriptional profiles have been acquired by reprogramming in response to chemotherapy in TNBC patients [165].

### 6.3. Immunotherapy and Single-Cell Sequencing: Overcoming the Barriers?

Given the high mutation rate of tumoral cells and vast intra-tumoral heterogeneity, targeting molecules and genes involed in signaling pathways of tumoral cells, cannot eliminate mutating cancer cells. However, the genetic instability of tumoral cells can give rise to tumoral neoantigens, paving the way for polyclonal expansion of tumor-infiltrating lymphocytes. Nevertheless, the immunosuppressive tumor microenvironment and inhibitory immune checkpoint axes inhibit the development of anti-tumoral immune responses. Thus, targeting programmed cell death protein 1 (PD-1) and cytotoxic T-lymphocyte-associated protein 4 (CTLA-4), as well-studied inhibitory immune checkpoints, have been considered one of the approaches to stimulate anti-tumoral immune responses. However, “heavy” reliance on these has been associated with an increased risk of immune-related adverse events development, especially among the prone patients [166]. In line with this, Louma et al. have studied the impact of the checkpoint blockade on immune cell populations in colitis. They reported a remarkable infiltration of CD8^+^ T cells with cytotoxic and proliferative features in colitis following the targeting of inhibitory immune checkpoints [167].

Besides the risk of autoimmunity development in the conventional form of immune checkpoints’ administration, the response rate of cancer patients to immune checkpoint inhibitors varies among the patients. Indeed, the safety issues and varied response rates between patients have posed serious questions about their future in cancer immunotherapy. However, recent advances in single-cell sequencing of immune cells and identifying the phenotype of tumor-infiltrated immune cells have shown promising results [168,169,170]. Durante et al. have indicated that the tumor-infiltrating CD8^+^ T cells in uveal melanoma mainly express the lymphocyte activation gene 3 (LAG3) rather than CTLA-4 and PD-1; this might explain the low response rates of affected patients to the current method of immune checkpoint inhibitors administration [171,172]. Furthermore, it has been reported that the expression of PD-1 and T cell immunoglobulin domain and mucin domain-3 (Tim-3) on T cells does not assure that anti-tumoral immune responses will be attenuated. Indeed, T cells positive for PD-1 and TIM-3 in lung tumors show a substantial level of proliferation and upregulation of effector transcriptional factors and produce proinflammatory cytokines [173]. Indeed single-cell sequencing of immune cells can categorize immune cells based on their expression of inhibitory immune checkpoints/signaling factors, which can help select immune checkpoint inhibitors and adjust their dosage [170]. Consistent with this, Sade-Feldman et al. have identified that TCF7 in the CD8^+^ T cells can be used as a prognostic marker for anti-PD-1 therapies and that its expression is associated with positive outcomes [169].

Therefore, the single-cell sequencing of immune cells and categorizing them based on inhibitory immune checkpoints/signaling factors can allow us to select immune checkpoint inhibitors and adjust their dosage, which can reduce the risk of autoimmunity development and increase response rates.

## 7. Conclusions

Although our knowledge of the oncogenic signaling pathways has increased over the past decade, tumor relapse and resistance to cancer therapy are still daunting challenges. Given the vast intra-tumoral heterogeneity obtained from single-cell sequencing of tumoral cells, eliminating tumor cells via targeting signaling pathways might be challenging. However, the intra-tumoral heterogeneity and genetic instability of tumoral cells can lead to the development of various neoantigens, which ultimately can pave the way for the infiltration of polyclonal lymphocytes into the tumor microenvironment. Nevertheless, the immunosuppressive tumor microenvironment and inhibitory immune checkpoints can substantially attenuate the anti-tumoral immune responses. Therefore, the data from single-cell sequencing of tumor-infiltrating lymphocytes can provide valuable insight into the inhibitory immune checkpoints/signaling factors in tumor-infiltrating lymphocytes, which can be used for selecting immune checkpoint inhibitors and adjusting their dosage for cancer patients. Although the single-cell sequencing of immune cells is in its infancy and further studies are needed for its translation into the clinics, the current evidence indicates that this approach can optimize the response rate of immune checkpoint inhibitors, minimize the related immune-related adverse events, and eliminate the mutating tumor cells.
